# Concurrent Chemotherapy of Malignant Glioma in Rats by Using Multidrug-Loaded Biodegradable Nanofibrous Membranes

**DOI:** 10.1038/srep30630

**Published:** 2016-07-29

**Authors:** Yuan-Yun Tseng, Yin-Chen Huang, Tao-Chieh Yang, Shun-Tai Yang, Shou-Cheng Liu, Tzu-Min Chang, Yi-Chuan Kau, Shih-Jung Liu

**Affiliations:** 1 Department of Neurosurgery, Shuang Ho Hospital, Taipei Medical University, Taipei, Taiwan; 2 Department of Surgery, School of Medicine, College of Medicine, Taipei Medical University, Taipei, Taiwan; 3 Department of Neurosurgery, Chang Gung Memorial Hospital, Chang Gung University College of Medicine, Tao-Yuan, Taiwan; 4 Department of Neurosurgery, Taichung Tzu Chi General Hospital, The Buddhist Tzu Chi Medical Foundation, Taichung, Taiwan; 5 Department of Mechanical Engineering, Chang Gung University, Tao-Yuan, Taiwan; 6 Department of Anesthesiology, Chang Gung Memorial Hospital, Tao-Yuan, Taiwan; 7 Department of Orthopedics, Chang Gung Memorial Hospital, Tao-Yuan, Taiwan

## Abstract

Glioblastoma multiforme has a poor prognosis and is highly chemoresistant. In this study, we implanted biodegradable 1,3-bis[2-chloroethyl]-1-nitroso-urea-, irinotecan-, and cisplatin-eluting poly[(d,l)-lactide-co-glycolide] (BIC/PLGA) and virgin nanofibrous membranes on the brain surface of C6 glioma-bearing rats in concurrent and virgin groups, respectively. The concentrations of all applied drugs were significantly higher in the brain than in the blood for more than 8 weeks in all studied rats. Tumor growth was more rapid in the vehicle-treated group, and tumor volumes were significantly higher in the vehicle-treated group. Moreover, the average survival time was significantly shorter in the vehicle-treated group (*P* = 0.026), and the BIC/PLGA nanofibrous membranes significantly reduced the risk of mortality (*P* < 0.001). Furthermore, the results suggested that the BIC/PLGA nanofibers reduced the malignancy of C6 glioma. The experimental findings indicate that the multianticancer drug (i.e., BIC)-eluting PLGA nanofibers are favorable candidates for treating malignant glioma.

Glioblastoma multiforme (GBM), the most aggressive malignant and most frequently occurring primary brain tumor, is currently incurable and has a poor prognosis^1^,^2^. Although intensive efforts have been exerted to identify an effective chemotherapy regimen for GBM in the past 30 years, the median survival of 12–15 months has not been appreciably improved^3^,^4^. Treatments for GBM include surgery, chemotherapy, and radiotherapy; however, GBM treatment remains difficult because contemporary curative treatments are unavailable. The primary objective of surgery is to remove as much of the tumor as possible without injuring the surrounding normal brain tissue required for normal neurological function. However, GBMs are surrounded by a zone of migrating, infiltrating tumor cells that invade the surrounding tissue, making it impossible to excise the entire tumor^5^. Surgery remains the main treatment in which the bulk of the tumor is removed, and the peripheral infiltrating zone is targeted by supplementary treatments such as radiotherapy and chemotherapy^2^,^6^,^7^.

Several new therapeutic approaches have been developed for GBM. However, these approaches have not increased the survival rate among patients with GBM. This therapeutic inadequacy can be attributed to the blood–brain barrier (BBB), which is related to the highly malignant characteristic of GBM. The BBB, which maintains a protective environment in the central nervous system (CNS), limits the delivery of chemotherapeutic agents to the brain tumor, and numerous drugs fail to achieve therapeutic concentrations at the tumor site, despite systemic toxicity having been achieved. Multiple attempts have been made to overcome the obstacle of the BBB. Conventional methods of enhancing drug concentrations in the brain, such as the disruption of the BBB, intraventricular drug injection, and local therapy, are highly invasive and are therefore inapplicable to long-term treatment regimens^8^. One of the most promising approaches for bypassing this barrier relies on the specific properties of nanoparticulate vectors designed to interact with BBB-forming cells at the molecular level; these approaches can be used for transporting drugs or other molecules without interfering with normal brain function^8^,^9^.

In addition to the limited intracerebral delivery of chemotherapeutic agents, another major cause of treatment failure is the resistance of primary tumors to chemotherapeutic agents^4^. Glioma arises within the confines of a variably intact BBB and is surrounded and preserved by functional brain tissue; it spreads diffusely beyond the gross tumor margin and is prone to chemotherapy resistance because of efflux and direct inactivation of drugs^10^. GBM is refractory to most cancer cytotoxic agents, and occasional responses are often short-lived because of the rapid development of resistance, which is a direct consequence of genetic transformation and tumor heterogeneity characteristics of GBM^11^. With rare exceptions, little success has been achieved with monotherapy in the treatment of human malignancy compared with treatment involving multiagent therapies. Therefore, a combinatorial drug approach is relevant within and among specific groups of drugs. New chemotherapeutic strategies have been used for preventing the development of tumor resistance by employing multiple adjuvant chemotherapeutic agents with differing antitumor mechanisms^4^,^12^.

To date, the use of chemotherapy in the treatment of malignant glioma has not yielded favorable results. Nitrosoureas, including lomustine (CCNU) and 1,3-bis[2-chloroethyl]-1-nitroso-urea (i.e., carmustine) (BCNU), administered as a monotherapy or multiagent therapy, are the most widely used chemotherapeutics for patients with GBM^13^. The active metabolite of these drugs is the chloroethyl carbonium intermediate, which reacts with the O^6^ position of guanine, resulting in the cross-linking of DNA strands, DNA duplication, and transcription impairment^14^. *In vitro*, BCNU exhibits antitumor activity against cell lines derived from various malignancies including leukemia, lung cancers, sarcomas, and gliomas^15^,^16^. Irinotecan is a camptothecin derivative that inhibits topoisomerase I, an essential nuclear enzyme required for the relaxation of supercoiled DNA, which is a topological change facilitating RNA transcription and DNA replication. In addition, irinotecan exhibits antitumor activity against human glioblastoma cells with multidrug resistance^17^,^18^. Cisplatin is a platinum complex with 2 chloride atoms and 2 amine groups positioned in the cis configuration. Once inside the body, the 2 chloride atoms are displaced by water molecules, resulting in a hydrated complex that crosslinks DNA strands and triggers programmed cell death. Cisplatin reduces AGAT activity *in vitro*; as have nitrosoureas, cisplatin has long been considered an active agent for the treatment of glioma^19^.

In the present study, we developed biodegradable multidrug (i.e., BCNU, irinotecan, and cisplatin)-loaded poly[(d,l)-lactide-co-glycolide] (BIC/PLGA) nanofibrous membranes by using the electrospinning technique. *In vivo* release characteristics of BCNU, irinotecan, and cisplatin into the brain parenchyma were evaluated. The BIC/PLGA nanofibrous membranes were implanted on the brain surface of C6 glioma-bearing rats to investigate the therapeutic efficacy of the membranes for GBM treatment. Serial brain magnetic resonance imaging (MRI) and regular histological examinations were also performed.

## Results

Nanofibrous membranes were successfully electrospun using appropriate process parameters, including the solvent, polymer concentration, and flow rate. Electrospun membranes were observed under a scanning electron microscope at a magnification of ×5000. The diameters of the electrospun drug-eluting PLGA nanofibers ranged from 375 to 1,200 nm, with a high porosity of the nanofibrous membranes.

### *In Vivo* Release Characteristics of Chemotherapeutic Agents from Nanofibers

**Figure 1:**
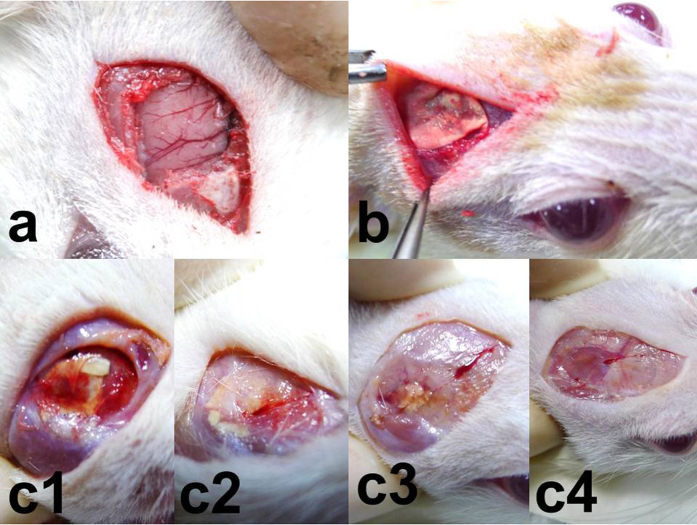
Surgical procedure and nanofibrous membrane degradation. (**a**) Craniectomy (approximately 1 × 1 cm) was performed. (**b**) Nanofibrous membranes (0.8 × 0.8 cm) were implanted on the brain surface of rats. (**c)** The nanofibrous membranes degraded gradually; **c1**: 2 weeks, **c2**: 4 weeks, **c3**: 6 weeks, and **c4**: 8 weeks. The nanofibrous membranes had nearly disappeared by 8 weeks.

**Figure 2:**
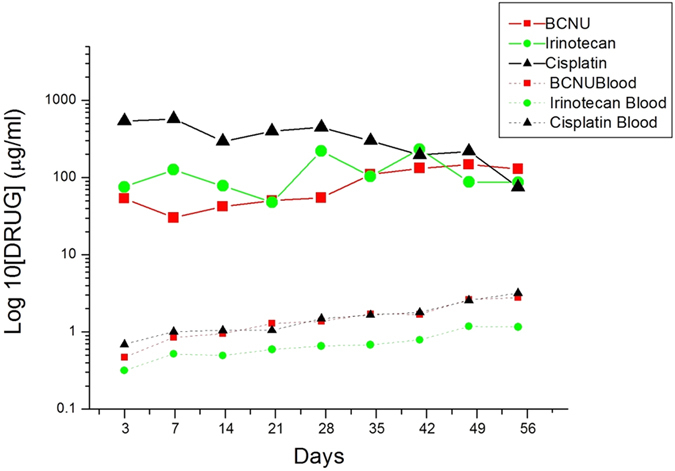
*In vivo* release curves for the release of BCNU, irinotecan, and cisplatin from the biodegradable nanofibrous membranes.

*In vivo* drug concentrations were determined for 8 weeks by using high-performance liquid chromatography. After excluding rats that died during the perioperative period (mainly because of anesthesia overdose or massive hemorrhage), exhibited brain injury, or evidenced brain, peritoneal, or systemic infection, at least 5 rats at each time point were enrolled for analysis of BCNU, irinotecan, and cisplatin concentrations. The BIC/PLGA nanofibrous membranes degraded gradually and were greatly diminished by the end of this study (Fig. 1c1). The *in vivo* release curves for the release of BCNU, irinotecan, and cisplatin from the biodegradable nanofibrous membranes are shown in Fig. 2. All 3 chemotherapeutic agents (i.e., BCNU, irinotecan, and cisplatin) were released rapidly from BIC/PLGA nanofibrous membranes. At Day 3, a high concentration of cisplatin was observed in the brain (539.72 ± 236.12 μg/mL), and the concentrations of all 3 chemotherapeutic agents were lower in the blood (0.47 ± 0.09 μg/mL, 0.31 ± 0.01 μg/mL, and 0.68 ± 0.03 μg/mL for BCNU, irinotecan, and cisplatin, respectively). The brain–blood drug concentration ratio reached the maximum value at Day 3 (118.94, 278.92, and 516.27 for BCNU, irinotecan, and cisplatin, respectively). The drug concentrations in the brain tissue remained high (approximately 100 μg/mL), and they were substantially higher in the brain than in the blood throughout the study period (8 wk). The drug concentrations in the blood increased slowly with the degradation of the PLGA nanofibers, reaching the maximum value at Day 56. The brain–blood concentration ratios of all drugs reached minimum values at Day 56 (27.91, 63.60, and 6.83 for BCNU, irinotecan, and cisplatin, respectively). The concentrations of all drugs were considerably higher in the brain than in the blood (systemic). At every time point, significant differences were observed in the concentrations of the chemotherapeutic agents between the brain and the plasma (*P* < 0.01).

### MRI

**Figure 3:**
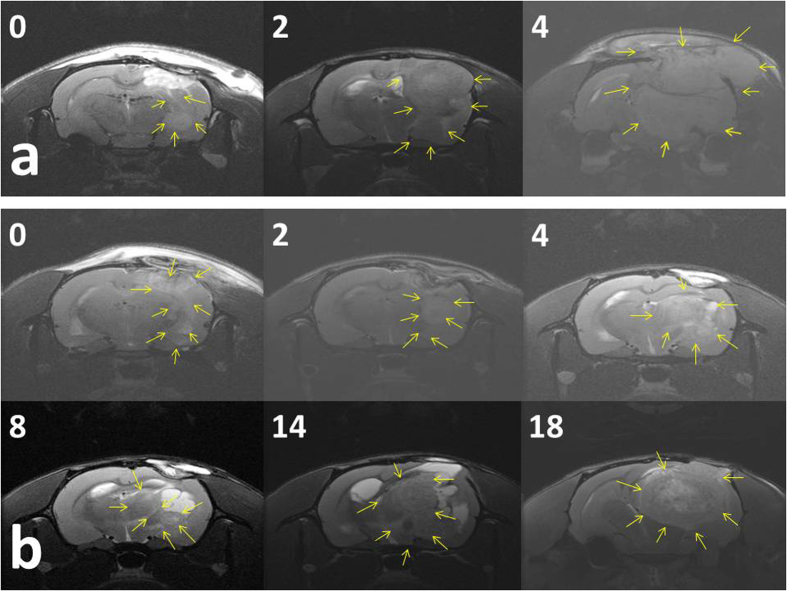
Serial rat brain MRI images of two rats. The number on the upper-left corner of each image indicates the number of weeks after nanofibrous membrane implantation. (**a**) The rat in the vehicle-treated group exhibited rapid tumor growth, leading to a severe mass effect. (**b**) The rat in the concurrent group evidenced temporary tumor stabilization; the tumor grew more slowly and reached the maximum tumor volume at 4 weeks; the tumor volume decreased between 4 and 8 weeks, and the tumor regrew progressively thereafter.

**Figure 4:**
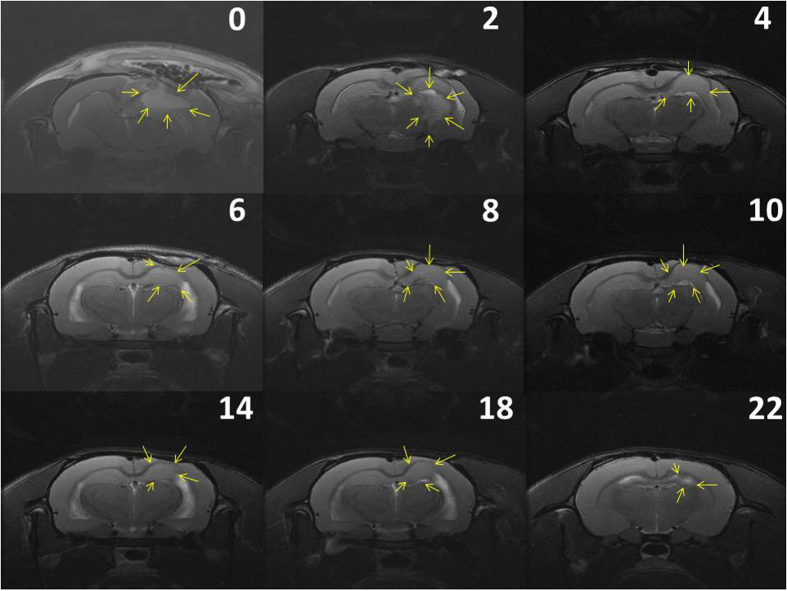
Serial brain MRI images of other rats in the concurrent group. The number on the upper-right corner of each image indicates the number of weeks after nanofibrous membrane implantation. The tumors grew slowly, and the maximum tumor volume was reached at Week 2; thereafter, the tumor volume decreased, evidencing a near-complete response at the end of this study (22 weeks).

Approximately 10–12 days after C6 glioma cell implantation on rat brains, T1- and T2-weighted images were obtained to ensure that glioma models were successfully established. Virgin (no drug loading) and BCNU-, irinotecan-, and cisplatin-eluting nanofibrous membranes were implanted on the brain surface of tumor-bearing rats in the vehicle-treated and concurrent groups, respectively. Serial brain MRI images were taken before membrane implantation and at 0, 2, 4, 6, 8, 10, 14, 18, and 22 weeks after implantation. The serial MRI images of the rats in the vehicle-treated group revealed that the implanted tumors grew rapidly, resulting in a severe mass effect (midline shift and brain stem compression) (Fig. 3a) and eventually causing death. The serial brain MRI images in Fig. 3b indicate that the implanted tumors grew more slowly in the concurrent group than in the vehicle-treated group; the tumor volume decreased between Weeks 4 and 8. Thereafter, the tumor progressively regrew, and the rats died at Week 23. The serial brain MRI images in Fig. 4 show that the initial tumor volume in the concurrent group (62.73 × 10^−3 ^mL) was similar to the mean tumor volume in the vehicle-treated group (60.36 ± 38.69 × 10^−3 ^mL); the tumor grew slowly, and the maximum tumor volume in the concurrent group was reached at Week 4. Thereafter, the tumor volume decreased steadily; the volume was greatly diminished by the end of this study.

**Figure 5:**
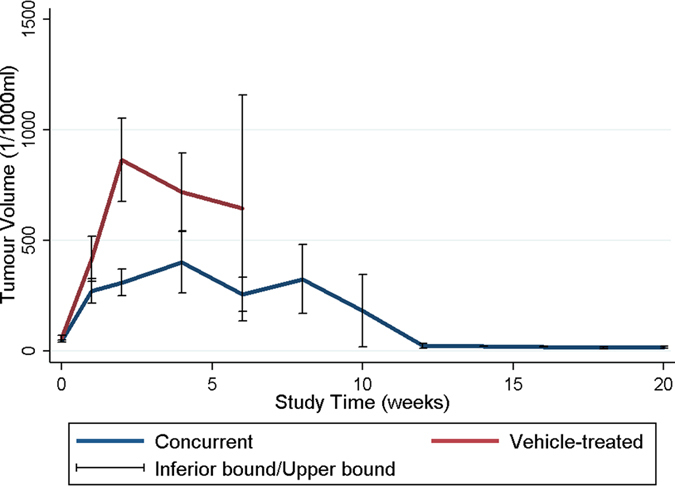
A repeated-measures mixed model was used to evaluate differences in the mean tumor volume between the 2 groups. The difference in mean tumor volume did not reach statistical difference initially, and the tumors grew more rapidly in the vehicle-treated group. Moreover, the mean tumor volume was significantly greater in the vehicle-treated group than in the concurrent group (*P* < 0.001).

The mean tumor volumes before membrane implantation (approximately 10–12 d after tumor cell implantation) were 60.36 ± 38.69 × 10^−3 ^mL in the vehicle-treated group and 46.32 ± 19.32 × 10^−3 ^mL in the concurrent group. The mean tumor volumes at various time points were high in the vehicle-treated group; however, the difference was statistically nonsignificant (*P* = 0.362). Moreover, the tumor volumes increased rapidly in the vehicle-treated group; the mean tumor volumes were 418.13 ± 396.10 × 10^−3 ^mL, 866.00 ± 498.52 × 10^−3 ^mL, 719.07 ± 430.94 × 10^−3 ^mL, and 647.07 ± 723.26 × 10^−3 ^mL at 1, 2, 4, and 6 weeks, respectively. By contrast, the tumor volumes increased at a slower rate in the concurrent group; the mean tumor volume at Week 1 was 172.05 ± 207.05 × 10^−3 ^mL, which was lower than that in the vehicle-treated group (*P* = 0.429). This finding was statistically nonsignificant. At Week 2, the mean tumor volume was significantly lower in the concurrent group than in the vehicle-treated group (866.00 ± 498.52 × 10^−3^ vs. 310.49 ± 207.18 × 10^−3 ^mL, *P* = 0.038). The tumor volumes increased slowly, and the maximum tumor volume in the concurrent group was reached at Week 4 (401.44 ± 460.62 × 10^−3 ^mL). After 4 weeks, the tumor volumes decreased; the mean tumor volume was 15.99 ± 7.88 × 10^−3 ^mL at the end of this study (after 16 wk). Figure 5 shows the differences in the mean tumor volumes between the vehicle-treated and concurrent groups, as evaluated using the repeated-measures mixed model. The tumor volumes increased rapidly in the vehicle-treated group, whereas the tumors slowly grew in first 4 weeks and decreased in volume thereafter in the concurrent group. A statistically significant difference was observed in the tumor volume between the 2 groups (*P* < 0.001).

### Survival Rate

**Figure 6:**
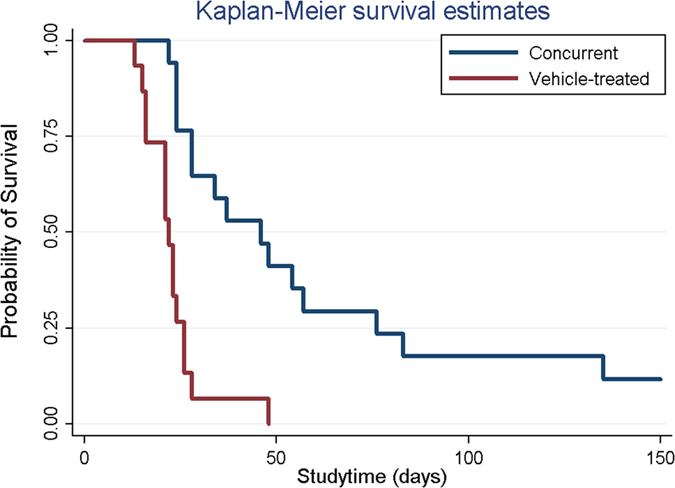
Overall survival of glioma-bearing rats in the concurrent and vehicle-treated groups. The survival time was significantly shorter in the vehicle-treated group (*P* = 0.026).

The rats that died during the perioperative period (within the first postoperative day) and those that exhibited wound infection or no glioma formation were excluded. Glioma models were successfully established in 32 rats (15 and 17 rats in the vehicle-treated and concurrent groups, respectively) and confirmed through brain MRI. In the vehicle-treated group, 14 rats died within 4 weeks after tumor cell implantation; only 1 rat survived for more than 4 weeks, dying at Day 48. The median survival time was 22.87 ± 8.21 and 60.00 ± 44.43 days for rats in the vehicle-treated and concurrent groups, respectively. The survival time was significantly shorter in the vehicle-treated group (*P* = 0.026). Figure 6 shows the Kaplan–Meier curves for representative survival times. In the concurrent group, the BIC/PLGA nanofibrous membrane significantly reduced the risk of mortality (*P* < 0.001).

### Pathology

**Figure 7:**
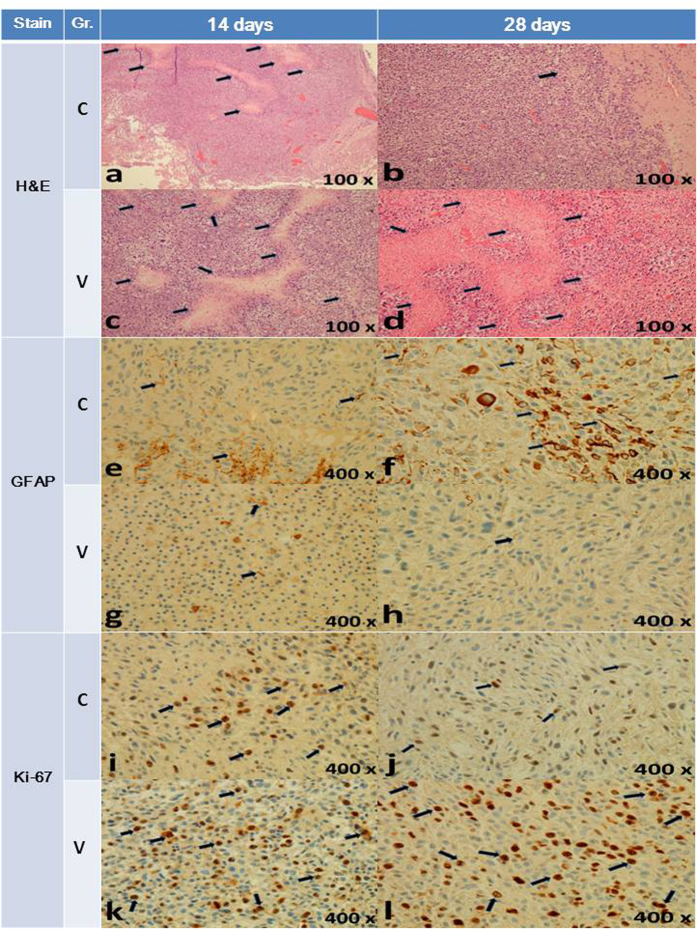
The black arrows indicate the central necrotic area (**a**–**d**), GFAP expression (**e**–**h**), and Ki-67-positive cells (**i**–**l**). The central necrotic area decreased in the concurrent group (**a**→**b**), whereas it increased in the vehicle-treated group (**c**→**d**). In the concurrent group, GFAP expression was suppressed at 14 days (**e**,**f**), and obvious GFAP expression was observed at 28 days (**f**). The Ki-67 index is 14.02% in (**i**), 8.92% in (**j**), 21.54% in (**k**), and 31.95% in (**l**). Gr.: group; C: concurrent group; V: vehicle-treated group; H&E: hematoxylin and eosin; GFAP: glial fibrillary acidic protein.

Figure 7 demonstrates the pathological examination results. According to the results of hematoxylin and eosin (H&E) staining, the tumor volume and necrotic area increased in the vehicle-treated group and decreased in the concurrent group. According to immunohistochemistry staining, glial fibrillary acidic protein (GFAP) expression was suppressed in the concurrent and vehicle-treated groups at Day 14. Obvious GFAP expression was observed at Day 28 in the concurrent group, whereas no GFAP expression was observed in the vehicle-treated group. The Ki-67 labeling index in the vehicle-treated group was 21.54% at Day 14, increasing to 31.95% at Day 28. By contrast, the Ki-67 labeling index was 14.02% at Day 14 and decreased to 8.92% at Day 28 in the concurrent group. Thus, the pathological examinations revealed an increased central necrotic area, loss of GFAP expression, and increased Ki-67 labeling index.

## Discussion

Surgery followed by standard radiotherapy with concomitant and adjuvant chemotherapy with temozolomide is the current standard treatment in malignant glioma patients aged <70 years; however, the prognosis of this disease remains poor^7^. Malignant gliomas originate from glial cells, which support nerve cells throughout the entire brain. GBMs have the characteristic abilities of infiltrating healthy brain tissue and forming satellite tumors. This capacity for migration makes their treatment exceedingly difficult, and they are invariably fatal. Even after resection, invasive cells can generate tumors within centimeters of the resection site. For other solid tumors, the clinical efficacy of cytotoxic chemotherapeutic agents is marginal for GBM, primarily because of systemic chemotherapy toxicities and drug delivery limitations^20^,^21^. Treatment failure results from several factors, including BBB limitation and high intratumoral pressure gradients, which restrict drug penetration^22^ and cause resistance to chemotherapeutic agents^4^.

Various approaches are currently employed with the aim of achieving effective local delivery with minimal systemic side effects, such as in the administration of therapeutic molecules via intracranially implanted catheters, convection-enhanced drug delivery, and controlled-release polymers^16^. The only interstitial chemotherapy treatment approved to date for malignant glioma is the Gliadel wafer, a biodegradable polymer containing 3.85% BCNU, which is placed in the resection cavity at the time of surgery^23^. The safety of this approach has been demonstrated in previous studies^24^,^25^. Gliadel wafers deliver a single chemotherapeutic agent to the brain cavity. Pharmacological studies have shown that most drug release from Gliadel wafers occurs in the first 5–7 days^26^. However, the cytotoxicity of BCNU is frequently compromised by the development of drug resistance, leading to subsequent tumor growth^27^. The survival of patients receiving Gliadel wafer treatment was approximately 2 months longer than that of patients who did not receive the treatment^25^,^28^. The therapeutic effect has yet to be improved to increase the survival time of patients with high-grade malignant glioma^29^.

Numerous promising biopharmaceutical agents have been developed; however, few of them (<5%) can be used for treating tumors in the CNS, as they cannot be delivered to the desired site of action in therapeutically relevant quantities because of the BBB^9^,^30^. Polymeric nanoparticles are nanosized carriers (1–1000 nm) that are composed of natural or synthetic polymers, in which the drug can be absorbed, chemically linked to the surface, or loaded in a solid state or solution. The current use of polymeric nanoparticles is one of the most promising approaches for drug delivery to the CNS^31^,^32^. Polymeric nanoparticles have several advantages, such as their high drug-loading capacity (i.e., ability to deliver a high number of drug molecules to cells)^33^ and nanoparticles protecting the embedded drugs against chemical or enzymatic degradation, which increase the likelihood of the active molecule reaching the target site^31^. The development of nanoparticles formulated from polylactic acid and PLGA offers several advantages for the delivery of therapeutic agents to the CNS. These polymers are biodegradable and biocompatible, and nanoparticles formulated from them do not induce any inflammatory response. In addition, their degradation products (i.e., glycolic acid and lactic acid) are eventually converted to carbon dioxide and water though the Krebs cycle and are ultimately eliminated^34^,^35^.

Another major cause of treatment failure is the resistance of primary tumors to chemotherapy^4^. Resistance is predictable considering the marked heterogeneity of tumor cells within and between individual tumors. One study related to GBM pathobiology and its clinical course proposed the specific targeting of more than one tumor compartment, further suggesting that one mechanism controls GBM progression^12^. Combinatorial therapy, or drug cocktails comprising multiple adjuvant chemotherapeutic agents with different antitumor mechanisms, has been introduced for preventing tumor resistance^4^,^12^,^36^. The efficacy of these agents as monotherapies is marginal; however, new multitargeting chemotherapeutic agents in combination with radiotherapy will likely play an increasingly crucial role in the management of GBM; several randomized prospective studies on this topic are ongoing^7^,^12^. Most multicenter randomized European (e.g., EORTC) and US (e.g., RTOG and NABTG) clinical trials are currently testing these new agents in combination with standard chemoradiotherapy (i.e., radiotherapy plus concomitant and adjuvant temozolomide) for comparison with standard chemoradiotherapy alone^7^. Alkylating agents are ideal candidates for combinatorial chemotherapy with irinotecan because they have different mechanisms of action and different organ toxicities. Irinotecan and BCNU appear to act synergistically against CNS cancer cell lines^14^,^37^. In a phase II trial, the combination of irinotecan with BCNU exhibited antitumor activity against recurrent or newly diagnosed malignant glioma that was comparable to the antitumor activity of irinotecan alone, but with an apparently increased toxicity^13^. Survival was significantly longer in patients receiving cisplatinum plus BCNU compared with that in patients receiving cisplatinum plus etoposide or carboplatinum plus BCNU, with a median survival time of 21.5, 15, and 15 months, respectively (log-rank test *P* = 0.01)^38^. Grossman *et al*. conducted a phase II study on the continuous infusion of BCNU and cisplatin followed by cranial irradiation, finding that this chemotherapy regimen appears to have significant antitumor activity and may prolong the survival of adults with newly diagnosed high-grade astrocytoma^39^. The Johns Hopkins Oncology Center treated 15 patients with GBM by applying the following regimen: concurrent BCNU (40 mg/sqm/die) and cisplatin (40 mg/sqm/die) for 3 days every 3–4 weeks and whole-brain irradiation with 45 Gy plus a 15-Gy boost to the preoperative volume. This sequential chemoradiotherapeutic regimen appears to have significant antitumor activity in adults with newly diagnosed high-grade gliomas^40^.

A higher drug concentration theoretically has a more efficient therapeutic effect and prevents tumor resistance. The high BCNU concentrations used for the treatment of brain tumors exhibit crucial biological consequences. *In vitro* studies have found that BCNU-resistant human glioma cells are effectively killed at a BCNU concentration of 250 μM (53.75 μg/mL)^41^. In our study, BCNU was released from the BIC/PLGA nanofibrous membrane at a high concentration for more than 8 weeks; the BCNU concentration ranged from 30.26 ± 5.49 to 147.80 ± 83.07 μg/mL. In addition to BCNU, cisplatin (range: 75.11 ± 16.47 to 576.01 ± 135.25 μg/mL) and irinotecan (range: 75.95 ± 14.93 to 221.45 ± 35.27 μg/mL) concentrations were extremely high. In contrast to the high concentrations in the brain tissue, the concentrations of BCNU (range: 0.47 ± 0.03 to 2.79 ± 0.02 μg/mL), irinotecan (range: 0.32 ± 0.04 to 1.16 ± 0.07 μg/mL), and cisplatin (range: 0.47 ± 0.04 to 2.35 ± 0.10 μg/mL) in the blood were relatively low. The high brain–blood ratio can minimize systemic side effects caused by chemotherapy, particularly the combination of concurrent (cocktail) multiple chemotherapeutic agents. Most chemotherapeutic agents should be administrated through intravenous infusion; the systemic toxicity limits treatment. Because of the potential lung toxicity of BCNU, lomustine has been used in a procarbazine, CCNU, and vincristine (PCV) regimen^42^,^43^. This regimen has been employed extensively for treating malignant glioma. Because of the high incidence of hematologic toxicities, the regimen is repeatedly modified (lomustine, 110 mg/m^2^ on Day 1; procarbazine, 60 mg/m^2^ on Days 8–21; and PCV, 1.4 mg/m^2^ on Days 8 and 29, with the cycle repeated every 6 weeks)^42^,^43^.

In this study, the survival rate in the concurrent group was significantly higher than in the vehicle-treated group. Most of the rats in the vehicle-treated group (14 of 15) died within 4 weeks because of the rapid enlargement of the C6 glioma. In the concurrent group, near-complete responses were observed in 2 rats; these rats survived, and their tumors had nearly disappeared by the end of this study. Ten rats among the 17 in the concurrent group survived for more than 4 weeks, and the serial brain MRI revealed temporary tumor stabilization. The tumors grew at a slower rate in the rats in the concurrent group than in those in the vehicle-treated group in the first 4 weeks; the maximum tumor volume in the concurrent group was reached by Week 4. The tumor volumes decreased subsequently between Weeks 4 and 8, and the tumors regrew progressively approximately 8 weeks later. Compared with that in the vehicle-treated group, the tumor growth rate in the concurrent group decreased significantly. Furthermore, pathological examination and immunohistochemical staining revealed that the tumor volume and central necrotic area decreased in the concurrent group. The loss of GFAP expression, frequently observed in high-grade astrocytoma, may represent the undifferentiated state of these cells. Malignant astrocytomas are often GFAP negative, and the loss of GFAP expression has been observed in numerous high-grade gliomas^44^. The proliferative index (i.e., Ki-67) is a potent biologic marker that estimates the growth of neoplasms quantitatively, thereby aiding in identifying the prognosis of patients with neoplasms. Certain studies have reported an inverse relationship between cell proliferation and patient survival, which is shorter for tumors with a high proliferation index and longer for tumors with a low index^45^. In the vehicle-treated group, the obvious suppression of GFAP expression and an elevated proliferative index indicated the progressively malignant biological behavior of glioma cells. Moreover, the increased GFAP expression and decreased Ki-67 ratio in the concurrent group confirmed that the malignancy of C6 glioma decreased after the implantation of the BIC/PLGA nanofibrous membrane^44^,^46^. The complete response rate was low in our study, and we believe that outcomes can be improved if tumors are resected before BIC/PLGA nanofibrous membrane implantation.

In conclusion, chemotherapy, previously considered to be of marginal benefit, was clearly demonstrated to produce an effect on the survival time, time to tumor progression, and quality of life in C6 glioma-bearing rats. Using the electrospinning technique, we employed multiple chemotherapeutic agents in developing a biodegradable BCNU-, irinotecan-, and cisplatin-eluting PLGA nanofibrous membrane. Investigating *in vivo* drug concentrations, we demonstrated that the BIC/PLGA nanofibers can release a high concentration of chemotherapeutic agents in brain tissue for more than 8 weeks without inducing the mass effect and inflammatory response. Serial MRI images of tumor-bearing rats implanted with BIC/PLGA nanofibrous membranes revealed a slower tumor growth rate compared with that in those implanted with virgin membranes. Pathological examinations evidenced decreased malignancy. The survival rate was significantly higher in rats implanted with BIC/PLGA nanofibrous membranes (i.e., the concurrent group). These results suggested that biodegradable electrospun BCNU-, irinotecan-, and cisplatin-eluting PLGA nanofibers are potential components of an interstitial chemotherapeutic strategy for GBM treatment.

## Materials and Methods

### Preparation of Drug-Eluting PLGA Nanofibrous Membranes

The commercially available PLGAs employed in this study were obtained in powder form (Resomer RG 503; Boehringer, Ingelheim, Germany), with a lactide–glycolide ratio of 50:50 and particle sizes ranging 100–200 μm. A differential scanning calorimeter (TA-2000; DuPont, USA) was used to measure the thermal properties of PLGA, revealing a PLGA glass transition temperature of approximately 46.5 °C. The chemotherapeutic agents employed in this study included BCNU, irinotecan, and cisplatin, all of which were purchased from Sigma-Aldrich (USA). The solvent hexafluoroisopropanol (HFIP) was additionally purchased from Sigma-Aldrich.

Drug-loaded nanofibrous membranes were prepared using the electrospinning technique^29^. The laboratory setup for electrospinning consisted of a hypodermic syringe needle connected to a high-voltage (17 kV) direct current power supply, a syringe pump, and a grounded collector. To prepare the nanofibrous membranes, PLGA (240 mg), BCNU (20 mg), irinotecan (20 mg), and cisplatin (20 mg) were first dissolved in 1 mL of HFIP (Sigma-Aldrich, USA). The polymer solution was then loaded into the syringe and extruded from the needle tip at a constant rate (1.8 mL/h), employing the syringe pump at room temperature. The distance between the needle tip and the ground collector was 12 cm. High voltage was applied to the solution droplet, charging the body of the solution. The electrostatic repulsion counteracted the surface tension, and the droplet was stretched; a stream of liquid erupted from the surface. The electrospun nanofibers were collected in nonwoven membrane form on the collector (i.e., an aluminum plate). The thickness of the electrospun membrane was approximately 0.11 mm.

### Surgical Procedure and Animal Care

All experimental procedures were approved by the Institutional Animal Care and Use Committee of Taipei Medical University (LAC-2013-0172) and were conducted in accordance with standard animal research practice. Fifty Wistar rats weighing 200–300 g were anaesthetized by administering an intraperitoneal injection of 6% chloral hydrate (0.6 mL/kg body weight). The rats were randomly divided into 9 groups on the basis of the sampling time points: 3 days and 1, 2, 3, 4, 5, 6, 7, and 8 weeks, with 5 or 6 rats per group. After sterilization and shaving, a 1–1.5-cm scalp incision was made in the postorbital region (between the eye and the ear). After the dissection of scalp fascia and muscle with a scalpel, a craniectomy (approximately 10 × 10 mm) was performed using an electric burr (Fig. 1a). After local hemostasis, the prepared thin BIC/PLGA nanofibrous membrane was implanted on the surface of the brain tissue (Fig. 1b). The scalp wound was sutured with 3–0 nylon. After surgery, the rats were randomly housed in cages, with 3 or 4 rats per cage. They were fed standard laboratory food and water. Rats with intraoperative brain injury or infection in the scalp, skull bone, or brain tissue were excluded from this study.

### *In Vivo* Pharmacokinetics of Chemotherapeutic Agents

The rats were intraperitoneally administered an overdose of anesthesia at more than 1.2 mL/kg bodyweight. Blood samples were collected using syringes through cardiac puncture. Additionally, ipsilateral brain tissue (covered by the BIC/PLGA nanofibrous membrane) was extirpated. The wedge-shaped brain tissue (8 × 8 mm with a thickness of 8–10 mm) at the brain surface was sliced into 5 layers (i.e., Layers 1–5 from the surface beneath the membrane down to the center of the brain, with each layer having a thickness of approximately 1.5 mm) by using a rodent brain slicer (Zivic Instruments, USA). Approximately 0.05 g of brain tissue was sampled from each layer. All specimens (blood and brain) were collected at Day 3 and at Weeks 1–8. The specimens were centrifuged, and the plasma was collected and stored at −80 °C until analysis. The drug concentrations in the specimens were determined using HPLC.

### Animal and Tumor Cell Implantation

Another 40 adult Wistar rats weighing 200–300 g were acclimatized for 1 week and caged in groups of 9, with 4 or 5 rats per group. For tumor cell implantation, animals were deeply anesthetized by administering an intraperitoneal injection of 6% chloral hydrate (0.6 mL/kg body weight) and were then placed in a stereotactic device (Leitz, Wetzlar, Germany). A 1.5-cm scalp incision was made in the postorbital region and craniectomy (approximately 10 × 10 mm) was performed using an electric burr. Tumor material from frozen stock was introduced into a tuberculin syringe (B. Braun, Melsungen, Germany) linked to a 21-gauge needle. The needle tip was placed into the right temporal lobe at a depth of approximately 0.5 cm. The C6 glioma cells (10 μL, approximately 10^6^ cells) were injected through an implanted catheter at a rate of 1.0 μL/min. The scalp incision was sutured with 3–0 nylon; the wound was flushed with iodinated alcohol.

These 40 rats were randomly divided into 2 groups, with 20 rats per group. A virgin PLGA membrane (0.8 × 0.8 cm) covering the brain surface was implanted in the vehicle-treated group, whereas a BIC/PLGA nanofibrous membrane (0.8 × 0.8 cm) was implanted in the concurrent group. After implantation, the scalp wound was sutured with 3–0 nylon. Rats with intraoperative brain injury and those that died within the first postoperative day or exhibited wound infection or no glioma formation were excluded from this study.

### MRI and Microscopic Examination

The gross wound appearances were observed daily, and brain MRI was performed regularly with a 7 Tesla Biospec MR imager (Bruker, Ettlingen, Germany). Before membrane implantation (approximately 10 d after C6 glioma cell implantation), T1- and T2-weighted images were obtained to ensure that the glioma models were successfully established and excluded epidural, subdural, and intracerebral hemorrhage. T2-weighted images were obtained as a reference to identify the tumor region 2, 4, 6, 8, 10, 14, 18, and 22 weeks after nanofibrous membrane implantation. The tumor volume was reconstructed and calculated using the open-source, FDA-approved Digital Imaging and Communication in Medicine imaging OsiriX software. After follow-up MRI, 1 to 2 rats in each group were sacrificed, and the brain tissues were removed carefully for pathological examination. The brain tissue was fixed in 10% formalin and embedded in paraffin. Coronal sections (6-μm thickness) were prepared and stained with H&E, GFAP, and Ki-67.

### Statistical Analyses

Data were expressed as mean ± standard deviation; *P* < 0.05 was considered significant. The data collected from the samples were analyzed using the paired sample *t* test; all analyses were performed using the commercially available Stata Version 12.0 software (Stata, College Station, TX). Survival curves were plotted using the Kaplan–Meier method, with statistical significance determined using the post hoc log-rank test. A repeated-measures mixed model was employed to evaluate the effect of different treatments on the growth of implanted tumor cells.

## Additional Information

**How to cite this article**: Tseng, Y.-Y. *et al*. Concurrent Chemotherapy of Malignant Glioma in Rats by Using Multidrug-Loaded Biodegradable Nanofibrous Membranes. *Sci. Rep.* **6**, 30630; doi: 10.1038/srep30630 (2016).

## References

[b1] Friedman H. S. . Bevacizumab alone and in combination with irinotecan in recurrent glioblastoma. J Clin Oncol 27, 4733–4740 (2009). 19720927 10.1200/JCO.2008.19.8721

[b2] Jain K. K. Role of nanobiotechnology in the personalized management of glioblastoma multiforme. Nanomedicine (London, England) 6, 411–414 (2011). 10.2217/nnm.11.12 21542679

[b3] Henson J. W. Treatment of glioblastoma multiforme: a new standard. Archives of neurology 63, 337–341 (2006). 16533960 10.1001/archneur.63.3.337

[b4] Affronti M. L. . Overall survival of newly diagnosed glioblastoma patients receiving carmustine wafers followed by radiation and concurrent temozolomide plus rotational multiagent chemotherapy. Cancer 115, 3501–3511 (2009). 19514083 10.1002/cncr.24398

[b5] Stummer W. . Extent of resection and survival in glioblastoma multiforme: identification of and adjustment for bias. Neurosurgery 62, 564–576, discussion 564–576 (2008). 18425006 10.1227/01.neu.0000317304.31579.17

[b6] Attenello F. J. . Use of Gliadel (BCNU) wafer in the surgical treatment of malignant glioma: a 10-year institutional experience. Ann Surg Oncol 15, 2887–2893 (2008). 18636295 10.1245/s10434-008-0048-2

[b7] Minniti G., Muni R., Lanzetta G., Marchetti P. & Enrici R. M. Chemotherapy for glioblastoma: current treatment and future perspectives for cytotoxic and targeted agents. Anticancer Res 29, 5171–5184 (2009). 20044633

[b8] Kreuter J. & Gelperina S. Use of nanoparticles for cerebral cancer. Tumori 94, 271–277 (2008). 18564616 10.1177/030089160809400220

[b9] Barbu E., Molnar E., Tsibouklis J. & Gorecki D. C. The potential for nanoparticle-based drug delivery to the brain: overcoming the blood-brain barrier. Expert Opin Drug Deliv 6, 553–565 (2009). 19435406 10.1517/17425240902939143

[b10] Leten C., Struys T., Dresselaers T. & Himmelreich U. *In vivo* and ex vivo assessment of the blood brain barrier integrity in different glioblastoma animal models. J Neuro-oncol 119, 297–306 (2014). 10.1007/s11060-014-1514-2 24990826

[b11] Stupp R., Hegi M. E., Gilbert M. R. & Chakravarti A. Chemoradiotherapy in malignant glioma: standard of care and future directions. J Clin Oncol 25, 4127–4136 (2007). 17827463 10.1200/JCO.2007.11.8554

[b12] Debinski W. Drug cocktail for effective treatment of glioblastoma multiforme. Expert Rev Neurotherapeutics 8, 515–517 (2008). 10.1586/14737175.8.4.515 18416653

[b13] Reardon D. A. . Phase 2 trial of BCNU plus irinotecan in adults with malignant glioma. Neuro-oncol 6, 134–144 (2004). 15134628 10.1215/S1152851703000413 PMC1871982

[b14] Brandes A. A. . Second-line chemotherapy with irinotecan plus carmustine in glioblastoma recurrent or progressive after first-line temozolomide chemotherapy: a phase II study of the Gruppo Italiano Cooperativo di Neuro-Oncologia (GICNO). J Clin Oncol 22, 4779–4786 (2004). 15570079 10.1200/JCO.2004.06.181

[b15] Carter S. K., Schabel F. M. Jr., Broder L. E. & Johnston T. P. 1,3-bis(2-chloroethyl)-1-nitrosourea (bcnu) and other nitrosoureas in cancer treatment: a review. Adv Can Res 16, 273–332 (1972). 10.1016/s0065-230x(08)60343-7 4563045

[b16] Bota D. A., Desjardins A., Quinn J. A., Affronti M. L. & Friedman H. S. Interstitial chemotherapy with biodegradable BCNU (Gliadel) wafers in the treatment of malignant gliomas. Ther Clin Risk Manag 3, 707–715 (2007). 18472995 PMC2376068

[b17] Vredenburgh J. J., Desjardins A., Reardon D. A. & Friedman H. S. Experience with irinotecan for the treatment of malignant glioma. Neuro-oncol 11, 80–91 (2009). 18784279 10.1215/15228517-2008-075 PMC2718962

[b18] Nakatsu S. . Induction of apoptosis in multi-drug resistant (MDR) human glioblastoma cells by SN-38, a metabolite of the camptothecin derivative CPT-11. Can Chemother Pharmacol **3** 9, 417–423 (1997). 10.1007/s002800050592 9054955

[b19] Brandes A. A. . First-line chemotherapy with cisplatin plus fractionated temozolomide in recurrent glioblastoma multiforme: a phase II study of the Gruppo Italiano Cooperativo di Neuro-Oncologia. J Clin Oncol 22, 1598–1604 (2004). 15117981 10.1200/JCO.2004.11.019

[b20] Brandes A. A. & Fiorentino M. V. The role of chemotherapy in recurrent malignant gliomas: an overview. Cancer investigation 14, 551–559 (1996). 8951359 10.3109/07357909609076900

[b21] Black P. M. Brain tumors. Part 1. N Engl J Med 324, 1471–1476 (1991). 1822669 10.1056/NEJM199105233242105

[b22] Jain R. K. Transport of molecules in the tumor interstitium: a review. Cancer Res 47, 3039–3051 (1987). 3555767

[b23] Westphal M., Ram Z., Riddle V., Hilt D. & Bortey E. Gliadel wafer in initial surgery for malignant glioma: long-term follow-up of a multicenter controlled trial. Acta Neurochir 148, 269–275 (2006). 16482400 10.1007/s00701-005-0707-z

[b24] Valtonen S. . Interstitial chemotherapy with carmustine-loaded polymers for high-grade gliomas: a randomized double-blind study. Neurosurg 41, 44–48, discussion 48–49 (1997). 10.1097/00006123-199707000-00011 9218294

[b25] Brem H. . Placebo-controlled trial of safety and efficacy of intraoperative controlled delivery by biodegradable polymers of chemotherapy for recurrent gliomas. The Polymer-brain Tumor Treatment Group. Lancet 345, 1008–1012 (1995). 7723496 10.1016/s0140-6736(95)90755-6

[b26] Brem H. & Gabikian P. Biodegradable polymer implants to treat brain tumors. J Control Release 74, 63–67 (2001). 11489483 10.1016/s0168-3659(01)00311-x

[b27] Bacolod M. D. . Mechanisms of resistance to 1,3-bis(2-chloroethyl)-1-nitrosourea in human medulloblastoma and rhabdomyosarcoma. Mol Can Therapeutics 1, 727–736 (2002). 12479369

[b28] Westphal M. . A phase 3 trial of local chemotherapy with biodegradable carmustine (BCNU) wafers (Gliadel wafers) in patients with primary malignant glioma. Neuro-oncol 5, 79–88 (2003). 12672279 10.1215/S1522-8517-02-00023-6 PMC1920672

[b29] Tseng Y. Y., Liao J. Y., Chen W. A., Kao Y. C. & Liu S. J. Sustainable release of carmustine from biodegradable poly[((D,L))-lactide-co-glycolide] nanofibrous membranes in the cerebral cavity: *in vitro* and *in vivo* studies. Expert Opin Drug Deliv 10, 879–888 (2013). 23289446 10.1517/17425247.2013.758102

[b30] Pardridge W. M. Drug targeting to the brain. Pharm Res 24, 1733–1744 (2007). 17554607 10.1007/s11095-007-9324-2

[b31] Tosi G., Costantino L., Ruozi B., Forni F. & Vandelli M. A. Polymeric nanoparticles for the drug delivery to the central nervous system. Expert Opin Drug Deliv 5, 155–174 (2008). 18248316 10.1517/17425247.5.2.155

[b32] Olivier J. C. Drug transport to brain with targeted nanoparticles. NeuroRx 2, 108–119 (2005). 15717062 10.1602/neurorx.2.1.108 PMC539329

[b33] Kreuter J. Nanoparticles–a historical perspective. Int J Pharmaceutics 331, 1–10 (2007). 10.1016/j.ijpharm.2006.10.021 17110063

[b34] Lu C. T. . Current approaches to enhance CNS delivery of drugs across the brain barriers. Int J Nanomed 9, 2241–2257 (2014). 10.2147/IJN.S61288 PMC4026551 24872687

[b35] Makadia H. K. & Siegel S. J. Poly Lactic-co-Glycolic Acid (PLGA) as Biodegradable Controlled Drug Delivery Carrier. Polymers (Basel) 3, 1377–1397 (2011). 22577513 10.3390/polym3031377 PMC3347861

[b36] Kunwar S. . Phase III randomized trial of CED of IL13-PE38QQR vs Gliadel wafers for recurrent glioblastoma. Neuro-oncol 12, 871–881 (2010). 20511192 10.1093/neuonc/nop054 PMC2940677

[b37] Castellino R. C. . Schedule-dependent activity of irinotecan plus BCNU against malignant glioma xenografts. Can Chemother Pharmacol 45, 345–349 (2000). 10.1007/s002800050050 10755324

[b38] Boiardi A. . Interstitial chemotherapy plus systemic chemotherapy for glioblastoma patients: improved survival in sequential studies. J Neuro-oncol 41, 151–157 (1999). 10.1023/a:1006119505170 10222435

[b39] Grossman S. A. . Phase II study of continuous infusion carmustine and cisplatin followed by cranial irradiation in adults with newly diagnosed high-grade astrocytoma. J Clin Oncol 15, 2596–2603 (1997). 9215830 10.1200/JCO.1997.15.7.2596

[b40] Dazzi C. . A sequential chemo-radiotherapeutic treatment for patients with malignant gliomas: a phase II pilot study. Anticancer Res 20, 515–518 (2000). 10769716

[b41] Bodell W. J., Bodell A. P. & Giannini D. D. Levels and distribution of BCNU in GBM tumors following intratumoral injection of DTI-015 (BCNU-ethanol). Neuro-oncol 9, 12–19 (2007). 17018699 10.1215/15228517-2006-014 PMC1828109

[b42] Stupp R. . Changing paradigms–an update on the multidisciplinary management of malignant glioma. The oncologist 11, 165–180 (2006). 16476837 10.1634/theoncologist.11-2-165

[b43] Kappelle A. C. . PCV chemotherapy for recurrent glioblastoma multiforme. Neurology 56, 118–120 (2001). 11148250 10.1212/wnl.56.1.118

[b44] Wilhelmsson U., Eliasson C., Bjerkvig R. & Pekny M. Loss of GFAP expression in high-grade astrocytomas does not contribute to tumor development or progression. Oncogene 22, 3407–3411 (2003). 12776191 10.1038/sj.onc.1206372

[b45] Wakimoto H. . Prognostic significance of Ki-67 labeling indices obtained using MIB-1 monoclonal antibody in patients with supratentorial astrocytomas. Cancer 77, 373–380 (1996). 8625247 10.1002/(SICI)1097-0142(19960115)77:2<373::AID-CNCR21>3.0.CO;2-Y

[b46] Chiesa-Vottero A. G., Rybicki L. A. & Prayson R. A. Comparison of proliferation indices in glioblastoma multiforme by whole tissue section vs tissue microarray. Am J Clin Pathol 120, 902–908 (2003). 14671979 10.1309/8UAU-KFK3-NBDM-VTNU

